# Vaginal Natural Orifice Transluminal Endoscopic Surgery (vNOTES) for Gynecological Procedures in Obese Patients: A Systematic Review

**DOI:** 10.3390/jcm14165713

**Published:** 2025-08-12

**Authors:** Aristotelis-Marios Koulakmanidis, Christos Vrysis, Dimitrios Zacharakis, Evangelia Kontogeorgi, Ioakeim Sapantzoglou, Charalampos Voros, Athanasios Gkirgkinoudis, Christos Damaskos, Nikolaos Garmpis, Gerasimos Tsourouflis, Stylianos Kykalos, Themos Grigoriadis, Stavros Athanasiou, Dimitrios Dimitroulis

**Affiliations:** 1 1st Department of Obstetrics and Gynaecology, Alexandra Hospital, National and Kapodistrian University of Athens, 115 28 Athens, Greece; 2 Hellenic Minimally Invasive and Robotic Surgery (M.I.R.S.) Study Group, Medical School, National and Kapodistrian University of Athens, 115 28 Athens, Greece; 3 Department of Emergency Surgery, Laiko General Hospital, 115 27 Athens, Greece; 4 NS Christeas Laboratory of Experimental Surgery and Surgical Research, Medical School, National and Kapodistrian University of Athens, 115 28 Athens, Greece; 5 Department of Surgery, Sotiria General Hospital, 115 27 Athens, Greece; 6 Second Department of Propedeutic Surgery, Laiko General Hospital, Medical School, National and Kapodistrian University of Athens, 115 28 Athens, Greece

**Keywords:** vNOTES, laparoscopy, obesity, overweight, gynecology

## Abstract

**Aim:** This study was conducted to determine the feasibility, safety, and clinical outcomes of the vaginal natural-orifice transluminal endoscopic surgery (vNOTES) approach in gynecology for obese patients. **Methods:** PubMed, Cochrane Library, and Google Scholar were searched, from inception to April 2025. A systematic review was performed following the PRISMA guidelines. Studies assessing the use of vNOTES for gynecological procedures in obese women were included. The quality of included articles was evaluated according to the Newcastle–Ottawa Scale. **Results:** The search yielded three retrospective cohort studies, one cross-sectional, and ten case series. The patients in the vNOTES group (n = 99) had statistically significant shorter operative times, reduced hospitalization, lower postoperative pain scores, fewer perioperative complications, and improved quality of life when compared to the laparoscopy group (n = 84). A study compared obese women to non-obese women undergoing vNOTES and found that operative times were longer in the obese group. Conversion to laparoscopy or laparotomy occurred in fewer than 5% of cases, and intraoperative and postoperative complication rates were low across all studies. **Conclusions:** vNOTES appears to be safe and potentially superior to other minimally invasive techniques. The small sample size of the case series and the lack of a sufficient number of comparative studies limit the strength of the conclusions.

## 1. Introduction

Gynecology encompasses a wide range of benign and malignant surgical conditions, which may present on either an emergency or elective basis. Access to the peritoneal cavity can be established via laparotomy, vaginal approach, conventional laparoscopy, and robotic-assisted techniques [1,2]. In contemporary clinical practice, the primary objective is to minimize perioperative morbidity and complication rates while optimizing cosmetic outcomes through minimally invasive methods.

The transvaginal approach offers several benefits comparable to conventional laparoscopy, such as shorter hospital stays, faster recovery, and reduced postoperative pain, while avoiding any abdominal incisions [1,3]. However, it manifests certain limitations compared to laparoscopic access, particularly regarding the extent of peritoneal cavity exploration [3]. Moreover, its feasibility is reduced in patients without pelvic organ prolapse or with significantly enlarged uteri. To combine the advantages of both vaginal and laparoscopic approaches, the vaginal natural-orifice transluminal endoscopy surgery (vNOTES) technique was developed [4]. vNOTES is a hybrid approach that allows access to the peritoneal cavity via a small posterior colpotomy through the vagina, a natural orifice, after which a multi-channel single-port device is introduced and a pneumoperitoneum is established. The procedure is then performed using standard laparoscopic instruments [5,6,7]. This approach combines all the well-documented benefits of minimally invasive surgery with the scarless advantage of a purely transvaginal entry [5].

Obesity, defined as a body mass index (BMI) greater than or equal to 30, according to the World Health Organization (WHO) affected one in eight individuals globally in 2022 [8]. It is a chronic disease with widespread systemic implications. In particular, in women, obesity is associated with infertility, abnormal uterine bleeding, endometrial hyperplasia, and increased endometrial cancer risk [9]. Obesity also significantly elevates the risk of perioperative complications, including vascular injury, hemorrhage, venous thromboembolism, and wound infections [10,11]. In laparoscopic surgery, the increased abdominal-wall thickness may impede the insertion and maneuverability of trocars and instruments, thereby prolonging operative time. When combined with the Trendelenburg position, this may contribute to substantial respiratory compromise [12].

The increasing prevalence of obesity and the perioperative challenges highlight the need for innovative, minimally invasive surgical techniques. This literature review aims to evaluate the role of the vNOTES approach in the management of gynecological surgical conditions in obese patients.

## 2. Methods

### 2.1. Protocol Registration

This review has been registered in PROSPERO, a database for prospectively registered systematic reviews. The registration number for this review is CRD420251055312.

### 2.2. Search Strategy

Two independent reviewers (AMK, CV) conducted a literature review using the Cochrane Central Register of Controlled Trials (CENTRAL), PubMed, and Google Scholar, covering the period from inception to April 2025. The search included Medical Subject Headings (MeSH) terms and the combination of the following terms: vaginal natural-orifice transluminal endoscopic surgery, obesity, overweight, and gynecology. The search was performed without using any filters.

### 2.3. Eligibility Criteria

Studies evaluating vNOTES for gynecological procedures in obese patients were reviewed based on their titles, abstracts, and full texts. Additionally, the references of all studies were examined for further citations.

Two reviewers (AMK, CV) independently assessed all articles to determine their eligibility for inclusion in the review process. This study focused on both retrospective and prospective observational studies. We excluded articles featuring non-gynecological procedures, reviews, Chinese-language manuscripts, and in vitro investigations.

This study follows the Preferred Reporting Items for Systematic Reviews and Meta-Analysis (PRISMA) guidelines [13]. Table S1a,b in the Supplementary Materials presents the comprehensive PRISMA 2020 Checklist.

### 2.4. Quality Assessment of the Included Studies

The Newcastle–Ottawa Scale (NOS) was used for the quality assessment of observational studies (case–control and cohort studies). A NOS score of 7 or higher indicated a low risk of bias. A score between 4 and 6 indicated a high risk of bias, whereas a score of 3 or lower indicated a very high risk of bias [14]. Disagreements regarding quality assessment were resolved through discussion with a third reviewer.

### 2.5. Data Extraction

Two authors (AMK, CV) independently extracted details, including the year of publication, hospital setting, study methodology, sample size, type of intervention, demographic characteristics, comorbidities, and outcomes. This study presents findings derived from both univariate and multivariate analyses.

## 3. Results

**Figure 1:**
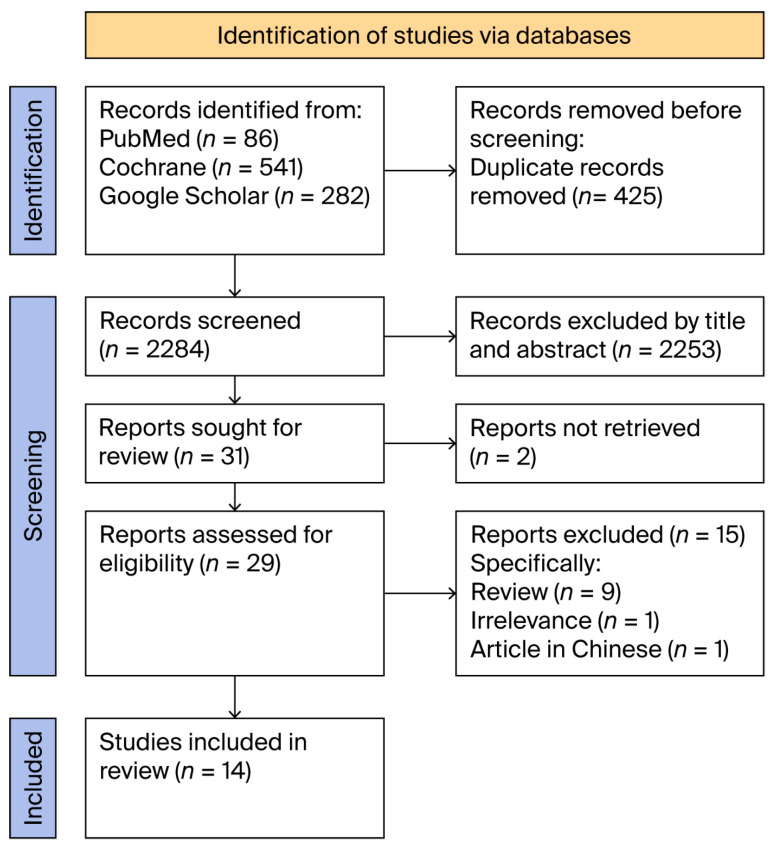
Flow diagram of identification, screening, and inclusion of relevant studies.

The relationship between the vNOTES approach in gynecological procedures and obesity was evaluated in four observational studies after a comprehensive literature review [15,16,17,18]. In addition, 10 case series provided further qualitative information about the clinical features of this condition [19,20,21,22,23,24,25,26,27,28]. Figure 1 presents the PRISMA flow diagram of the process of identification, screening, and selection of articles for inclusion in this analysis.

### 3.1. Case Series

Table 1 presents data regarding ten case series with 324 obese patients who underwent vNOTES gynecological procedures. These studies were conducted in tertiary-care hospitals in Turkey, Belgium, Spain, Russia, Singapore, Australia, and Switzerland between 2017 and 2024. The patient demographics varied, with BMI ranging from 28.5 to 60 kg/m^2^ and mean ages between 36.2 and 74.5 years. The most common procedure performed was the vNOTES hysterectomy, often combined with bilateral salpingo-oophorectomy, primarily for treating early-stage endometrial cancer, endometrial hyperplasia, postmenopausal bleeding, or benign uterine conditions. The operative time ranged from 82 to 233 min, with fewer than 5% of patients requiring conversion to laparoscopy or laparotomy. Hemoglobin loss ranged from 0.3 to 2.4 g/dL, and the hospital stay was typically short, with most patients discharged within one to two days. Postoperative pain was minimal, with visual analog scale (VAS) scores ranging from 1.0 to 3.5 within 6 to 48 h. Intraoperative and postoperative complications were low across all studies, with notable events including bladder injury, ovarian vessel bleeding, and seroma formation.

### 3.2. Comparative Studies

Table 2 presents four comparative observational studies conducted between 2017 and 2024 in tertiary-care hospitals in China, France, and Turkey. Among these studies, three studies were designed as retrospective cohorts, while one was cross-sectional. A total of 99 obese patients who underwent vaginal natural-orifice transluminal endoscopic surgery (vNOTES) and 84 control patients who underwent multi-port laparoscopic surgery (MPLS) or total laparoscopic hysterectomy (TLH) were included in the analysis. Across all studies, vNOTES was associated with statistically significant shorter operative times and shorter hospital stays. Additionally, patients who underwent vNOTES experienced statistically significant lower postoperative pain scores at both 6 and 24 h. This group also demonstrated fewer perioperative complications and reported improved quality-of-life scores. Conversion to conventional laparoscopy was required in selected cases due to intraoperative difficulties or anatomical limitations. The number of conversions per study is summarized in Table 2, and the specific reasons are detailed in the corresponding footnotes. Notably, the demographic characteristics of patients in both groups did not show significant differences.

Bouchez et al. (2023) retrospectively compared 54 obese to 146 non-obese patients who had undergone vNOTES hysterectomy [16]. The results revealed that the obese patients had statistically significant longer operative times (*p* < 0.001) than the control group. Table 3 summarizes the risk-of-bias assessment for the included studies; one study was classified as low-risk (NOS score ≥ 7), while the remaining three were considered high-risk (NOS score < 7).

## 4. Discussion

In the present study, the aim was to evaluate the role of vNOTES in gynecologic procedures among obese patients through a comprehensive review of the current literature. A total of 14 studies comprising 477 patients who underwent vNOTES for either benign or malignant indications were included. The most commonly performed operation was vNOTES hysterectomy, frequently in combination with bilateral saplingo-oophorectomy. The technique was associated with low rates of intraoperative and postoperative complications and demonstrated statistically significant improvements in perioperative outcomes compared to traditional laparoscopic approaches.

Several studies have investigated the potential benefits of the vNOTES approach compared to alternative surgical techniques across all BMI categories. In a randomized controlled trial (RCT), Baekelandt et al. documented significantly shorter operative time, reduced hospital stays, and lower postoperative pain levels in the vNOTES hysterectomy group compared to the total laparoscopic hysterectomy (TLH) group [7]. Similarly, Kaya et al. reported comparable findings, although no statistically significant difference was observed between the two groups in terms of postoperative visual analog scale (VAS) scores [15]. Additionally, Nef et al. confirmed the safety and feasibility of the procedure in elderly patients, a distinct subgroup characterized by a high prevalence of comorbidities [29]. Finally, three systematic reviews concluded that while both vNOTES hysterectomy and TLH are equally effective for benign indications, vNOTES is associated with shorter operative time, decreased length of hospital stay, and lower intraoperative blood loss [30,31,32].

Favorable outcomes associated with its application have positioned vNOTES as a valuable addition to the surgical field. Two separate meta-analyses by Steinemann et al. and Yang et al. reported lower postoperative pain scores, reduced rates of surgical-site infections, improved cosmetic outcomes, and shorter operative time and hospital stay with vNOTES compared to conventional laparoscopic approaches [32,33]. As a novel minimally invasive technique, vNOTES has attracted the interest of gynecologic oncologists. In this context, Lee et al. conducted a pilot study involving three patients, demonstrating the safety and feasibility of vNOTES for staging surgery in early endometrial cancer [34]. However, one of the main challenges in performing lymphadenectomy via vNOTES, compared to laparoscopic or robotic surgery, was the limited visualization of the retroperitoneal area and the lack of appropriate instruments for exposure and hemostasis [35,36]. This obstacle was addressed by Baekelandt et al., who described a new retroperitoneal vNOTES technique enabling sentinel lymph-node resection, and by Huber et al., who conducted a preliminary study with seven patients, showing the feasibility and safety of the method [37,38]. According to Deng et al. and Baekelandt et al., the application of vNOTES for the treatment of stage I endometrial cancer and identification of the sentinel lymph node appeared to be both safe and effective [38,39].

This study illustrated that the adoption of the vNOTES approach in obese patients yielded comparable outcomes to those observed in non-obese women when compared to conventional laparoscopy. Kaya et al. reported that the shorter operative time associated with vNOTES may have been attributable to easier access to the uterine pedicles and improved uterine manipulation. Furthermore, in obese patients, the increased amount of internal adipose tissue can hinder colpotomy and its closure during TLH. In contrast, with vNOTES, these steps are performed similarly to in a conventional vaginal hysterectomy [4]. Burnett et al. highlighted the easier handling of instruments during vNOTES procedures, as these did not need to traverse significant thickness of subcutaneous fat in obese patients undergoing laparoscopy [20]. Additionally, the retroperitoneal vNOTES approach eliminated the need for a steep Trendelenburg position and allowed for the use of lower pneumoperitoneum pressures, offering significant advantages in minimizing respiratory compromise in this patient subgroup [19,39]. Kale et al. and Kaya et al., in their respective analyses, attributed the reduced postoperative pain and decreased analgesic requirements associated with vNOTES to its scarless nature and the absence of abdominal-wall trauma or nerve injury. Consequently, these factors may have contributed to earlier patient mobilization and faster postoperative recovery [4,6].

The application of vNOTES in low-resource settings offers benefits, including quicker recovery and shorter hospital stays, which are valuable where healthcare structures are limited. However, success depends on the availability of surgical expertise and appropriate equipment. Efforts to expand vNOTES access should focus on training models and protocols adjusted to local constraints. As mentioned by Baekelandt et al. (2021), structured training and standardization are essential to ensure the reproducibility, safety, and adoption of this technique [40].

In addition, comparison between v-NOTES and vaginal hysterectomy remains of clinical interest. A recent systematic review and meta-analysis evaluated use of these two techniques in the general population [41]. However, there is limited evidence regarding these methods in obese patients. Since this review focuses on the outcomes of v-NOTES in individuals with obesity, it does not address this comparison. Future studies specifically designed to evaluate these two approaches in obese populations could provide additional clinical insights.

Although this study did not specifically analyze the learning curve associated with v-NOTES, it remains an important consideration, since adopting a new technique typically requires a period of skill development. Unfortunately, the current literature does not provide sufficient data to assess the learning curve, particularly regarding comparisons of operative times, complication rates, and procedural consistency during various phases of adoption. Future research that includes these factors could yield valuable insights.

It is important to acknowledge several limitations. There was a notable risk of publication and selection bias because this analysis primarily consisted of case series with small sample sizes, and the number of comparative studies that included a control group was limited. Additionally, heterogeneity existed among study designs, patient demographics, and surgical indications, which may impact the generalizability of the findings. Future research should prioritize large multicenter controlled trials that compare vNOTES with conventional methods and assess long-term outcomes, including recovery time and quality of life. Moreover, evidence-based studies are needed to better understand patient-reported outcomes, cost-effectiveness, and the learning curve for operators.

## 5. Conclusions

vNOTES appeared to be a promising minimally invasive surgical technique, especially in obese patients. While early outcomes are encouraging, the current evidence is limited due to small and heterogeneous studies. Future research should focus on large multicenter studies to validate these findings and determine the role of vNOTES in clinical practice.

## Figures and Tables

**Table 1:** Characteristics of the observational studies assessing vaginal natural-orifice transluminal endoscopic surgery (vNOTES) for gynecological procedures in obese patients.

First Author, Year of Publication, Reference #	Country	Hospital Setting, Study Period	Study Design	Intervention	Sample Size (Cases/Controls)	Patient Characteristics (Cases/Controls)	Outcomes ^c^
Wang F. et al., 2025 [17]	China	Tertiary-care hospital, January 2021 to June 2024	Retrospective cohort study	Ovarian tumor resection (vNOTES versus MPLS) ^a^	35 obese patients underwent vNOTES/41 obese patients underwent MPLS	Cases BMI, mean ± SD: 31.8 ± 2.3 Age, mean ± SD: 32.1 ± 6.5 y Controls BMI, mean ± SD: 32.4 ± 2.9 Age, mean ± SD: 31.7 ± 7.2 y	Shorter operative times (*p* < 0.05) Shorter hospital stays (*p* < 0.05) Fewer postoperative complications (*p* < 0.05) Lower VAS scores at 48 h post-operation (*p* < 0.05) Higher SF-36 ^e^ scores at 3 months post-operation (*p* < 0.05)
Bouchez M.-C. et al., 2023 [16]	France	Tertiary-care hospital, February 2020 to January 2022	Retrospective cohort study	vNOTES hysterectomy	54 obese patients/146 non-obese patients	Cases BMI, median (range): 34 (31.7–37.0) Age, mean ± SD: 47.1 ± 6.5 y Controls BMI, median (range): 24.3 (21.8–26.7) Age, mean ± SD: 47.3 ± 7.9 y	Longer operative time (*p* < 0.001) Concurrent procedure rate ^d^ (*p* = 0.031) Longer hospital stay (*p* = 0.032)
Kaya C. et al., 2021 [15]	Turkey	Tertiary-care hospital, January 2015 to December 2020	Cross-sectional study	Hysterectomy for benign gynecological conditions (vNOTES versus TLH) ^b^	48 obese patients underwent vNOTES/35 obese patients underwent TLH	Cases BMI, median (range): 31.9 (30–54.6) Age, median (range): 52 (40–74) y Controls BMI, median (range): 31.6 (30–42.2) Age, median (range): 49 (40–71) y	Lower 6th-hour VAS pain score (*p* = 0.003) Lower 24th-hour VAS pain score (*p* = 0.004) Shorter median time of operation (*p* < 0.001)
Matak L. et al., 2024 [18]	Croatia	Tertiary-care hospital, January 2022 to December 2023	Retrospective cohort study	Hysterectomy for benign gynecological conditions (vNOTES versus TLH) ^f^	16 obese patients underwent vNOTES/8 obese patients underwent TLH	Cases BMI, mean (range): 35.9 (31.2–52.0) Age, mean (range): 55 (45–77) y Controls BMI, mean (range): 34.1 (30.1–44.6) Age, mean (range): 57.5 (40–75) y	Shorter operative times (*p* < 0.05)

Abbreviations: vNOTES, vaginal natural-orifice transluminal endoscopic surgery; MPLS, multi-port laparoscopic surgery; BMI, body mass index; VAS, visual analog scale; SF-36, short form; SD, standard deviation. ^a^ Exclusion criteria: patients who identify as asexual; individuals experiencing vaginal stenosis; patients suffering from vaginal infections; individuals with a history of pelvic surgery; patients with ovarian cysts exceeding 10 cm; potential cases of pelvic adhesions; potential cases of cancer; and pregnancy. ^b^ Exclusion criteria: active urinary tract or pelvic infections; pregnancy; endometriosis; gynecological malignancy; history of pelvic radiotherapy; any contraindication of Trendelenburg position. ^c^ If a study included results from both univariate and multivariate analyses, we included only the results of the multivariate analysis. ^d^ Concurrent procedures: adhesiolysis; ureterolysis; prolapse procedure; ovarian cystectomy; trans obturator tape procedure. ^e^ The Short Form (36) Health Survey is a 36-item, patient-reported survey of patient health. ^f^ Exclusion criteria: stage II + prolapse requiring hysterectomy, hysterectomy due to endometriosis, subtotal hysterectomy, history of rectal surgery, suspected endometriosis of the rectovaginal septum, suspected malignancy, history of pelvic inflammatory disease (PID) and tubo-ovarian abscess, active pelvic inflammatory disease (e.g., chlamydia and gonorrhea), and pregnancy.

**Table 2:** Characteristics of the case series assessing vaginal natural-orifice transluminal endoscopic surgery (vNOTES) for gynecological procedures in obese patients.

First Author, Year of Publication, Reference #	Country, Hospital Setting, Study Period	Sample Size, Patient Characteristics	Intervention	Exclusion Criteria	Outcomes
Mat E. et al., 2021 [19]	Turkey, tertiary-care hospital, January 2019 to June 2019	Cases (*n* = 6) BMI, mean ± SD: 51.4 ± 6.1 Age, mean ± SD: 53.8 ± 7.5 y	Hysterectomy and bilateral salpingo-oophorectomy for early-stage type-1 endometrial cancer (T1aN0M0)	Any contraindication for pneumoperitoneum, dorsal lithotomy, Trendelenburg position or general anesthesia, the presence of sepsis, serious renal failure, severe cardiopulmonary disorder or coagulation disorders, the obliteration of the pouch of Douglas (history of endometriosis, pelvic inflammatory disease and diverticulitis), any laparotomy or laparoscopy involving the sigmoid or the rectum	Operating time, mean ± SD: 223.3 ± 25.6 min. Hemoglobin loss on postop day 1, mean ± SD: 1.5 ± 0.2 g/dL Length of hospital stay, mean: 2 days Postop VAS pain scores at 6, 24 h, mean: 3.16, 0.33 No conversion to conventional laparoscopy or laparotomy
Burnett A.-F. et al., 2024 [20]	Belgium, tertiary-care hospital, 2017 to 2023	Case group 1 (*n* = 84) BMI, mean (range): 45.7 (40–49.6) Age, mean (range): 49.6 (26–73) y Case group 2 (*n* = 19) BMI, median (range): 54.3 (50–62) Age, mean (range): 55.7 (35–72) y	Case group 1 (*n* = 84) VANH + BSO (*n* = 60), VANH (*n* = 6), vNOTES SO (*n* = 7), VANH, BSO, pelvic nodes (*n* = 5) Case group 2 (*n* = 19) VANH + SO (*n* = 16), vNOTES SO (*n* = 2)	Obliteration of the pouch of Douglas (history of endometriosis, pelvic inflammatory disease and diverticulitis), any laparotomy or laparoscopy involving the sigmoid or the rectum, adherence of the rectosigmoid to the posterior uterus	Case group 1 (*n* = 84) Operating time, median (range): 82 (30–232) min. Blood loss, median (range): 80 (10–400) mL Conversion to conventional laparoscopy or laparotomy (*n* = 6) ^a^ Case group 2 (*n* = 19) Operating time, median (range): 82 (30–232) min. Blood loss, median (range): 80 (10–400) mL No conversion to conventional laparoscopy or laparotomy (*n* = 1) ^b^ Postoperative vaginal cuff hematoma (*n* = 1)
Altintas M.-M. et al., 2022 [21]	Turkey, university hospital, April 2020 to January 2021	Cases (*n* = 6) BMI, mean ± SD: 44.3 ± 3.2 Age, mean ± SD: 47.7 ± 2.4 y	vNOTES hysterectomy ^c^ and concomitant umbilical hernia repair	Clinical diagnosis of renal, hepatic, hematologic, or neurologic disease, or malignant tumor	Operating time, mean ± SD: 88 ± 12.8 min. Length of hospital stay, mean: 2 days Hemoglobin loss, mean ± SD: 0.37 ± 0.26 g/dL Length of hospital stay, mean: 2 days Postop VAS pain scores at 8, 12, and 24 h, mean: 2.6, 1.6, and 1.2. No complication occurred intraoperatively Postoperatively: seroma (*n* = 1)
Guevara R. et al., 2024 [22]	Spain, tertiary-care hospital	Cases (*n* = 4) BMI, mean ± SD: 47.2 ± 4.8 Age, mean ± SD: 74.5 ± 3.9 y	vNOTES hysterectomy ^c^ for early-stage endometrial cancer	NR	Operating time, mean ± SD: 104.2 ± 23.9 min. Length of hospital stay, mean: 24 h No conversion to conventional laparoscopy or laparotomy No intraoperative complications or medium-term postoperative complications
Kale A. et al., 2022 [23]	Turkey, university hospital, January 2019 to April 2021	Case group 1 (*n* = 55) BMI, mean ± SD: 36.2 ± 4.3 Age, mean ± SD: 50 ± 5.6 y Case group 2 (*n* = 26) BMI, mean ± SD: 41.5 ± 9.7 Age, mean ± SD: 59.4 ± 8.0 y	Case group 1 (*n* = 55) ^c^ VANH, BSO (*n* = 43), vNOTES SO (*n* = 7), vNOTES SO + PFA + omentectomy (*n* = 4), Tubal ligation (*n* = 1) Case group 2 (*n* = 26) ^d^ VANH, BSO (*n* = 22), SO + omentectomy + PFA (*n* = 4)	NR	Case group 1 (*n* = 55) Operating time, mean ± SD: 64.2 ± 17.3 min. Hemoglobin loss, mean ± SD: 1.2 ± 0.6 g/dL Length of hospital stay, mean: 1 day Postop VAS pain scores at 6, 12, and 24 h, mean: 3.2, 1.1, and 0.7 No conversion to conventional laparoscopy or laparotomy Case group 2 (*n* = 26) Operating time, mean ± SD: 88.9 ± 61.4 min. Hemoglobin loss, mean ± SD: 0.7 ± 0.4 g/dL Length of hospital stay, mean: 1 day Postop VAS pain scores at 6, 12, and 24 h, mean: 3.3, 1.7, and 1.0 No conversion to conventional laparoscopy or laparotomy Intraoperatively: bladder injury (*n* = 1) Postoperatively: blood transfusions (*n* = 1)
Kapurubandara S. et al., 2025 [24]	Australia, university hospital, 2019	Cases (*n* = 20) BMI, median (range): 33.5 (27.8–38.3) Age, median (range): 51.5 (47–57) y	VANH + BSO + cystoscopy for complex hyperplasia with atypia (12/20, 60%), AUB (5/20, 25%) or prolapse with a concurrent ovarian mass (1/20, 5%)	NR	Operating time, median (range): 149 (138–198) min Blood loss, median (range): 125 (100–200) mL Length of hospital stay, median (range): 1.4 (1–2) days Conversion to conventional laparoscopy (5/20, 25%) ^e^ Intraoperatively, suture material was visible under the bladder mucosa at cystoscopy (*n* = 1) Twelve days postoperatively, vaginal bleeding was noted (*n* = 1)
Matak L. et al., 2024 [25]	Turkey, tertiary-care hospital, August to October 2023	Cases (*n* = 4) BMI, mean (range): 28.5 (25.4–34.6) Age, mean (range): 67 (53–82) y	VANH, BSO, pelvic lymphadenectomy, SLN biopsy for endometrial carcinoma	NR	Operating time, mean (range): 169 (150–200) min Hemoglobin loss (%), mean (range): 14 (9–20)% Length of hospital stay, mean (range): 2 days No intraoperative or postoperative complications
Ng W. et al., 2024 [26]	Singapore, tertiary-care hospital, 2024	Case 1 BMI: 57 kg/m^2^ Age: 52 y Case 2 BMI: 60.4 kg/m^2^ Age: 47 y	Case 1 vNOTES hysterectomy for postmenopausal bleeding Case 2 vNOTES hysterectomy for endometrial carcinoma	NR	Case 1 Operating time: 4 h and 30 min Blood loss: 500 mL Length of hospital stay: 5 days Postoperatively: PAF, starvation ketosis No conversion to conventional laparoscopy or laparotomy Case 2 Operating time: NR Blood loss: 150 mL Length of hospital stay: 2 days Postoperatively: PAF, starvation ketosis No conversion to conventional laparoscopy or laparotomy
Musin I.-I. et al., 2024 [28]	Russia, tertiary-care hospital, November 2022 to November 2023	Cases (*n* = 19) BMI, mean (range): 33.8 (27.6–35.2) Age, mean (range): 52 (47–62)	vNOTES hysterectomy for recurrent endometrial hyperplasia	History of retrocervical endometriosis or suspected obliteration of the posterior vaginal fornix, vaginal stenosis, presence of decompensated somatic comorbidities	Operating time, mean (range): 85 (64–110) min Blood loss, mean (range): 147 (120–180) mL Length of hospital stay, mean (range): 3 (3–5) days Postop VAS pain scores at 24, 48, and 72 h, mean: 3.0, 2.0, and 1.0 No conversion to conventional laparoscopy or laparotomy No intraoperative or postoperative complications
Hurni Y. et al., 2023 [27]	Switzerland, tertiary-care hospital, May 2020 to April 2023	Cases (*n* = 79) BMI, median (range): 35.2 (30.1–49.4) Age, median (range): 51 (32–79) y	vNOTES hysterectomy (*n* = 52), vNOTES SO (*n* = 26), and vNOTES myomectomy (*n* = 1)	NR	Operating time (hysterectomy), median (range): 91 (44–193) min Operating time (SO), median (range): 51 (18–56) min Length of hospital stay, median: 2 days Postop VAS pain scores at 12, 24 h, and 48 h, mean: 1.0, 2.0, and 1.0 Conversion to conventional laparoscopy (*n* = 4) ^f^ Intraoperatively: bladder injury (*n* = 10, rectal serosal tear (*n* = 2) Postoperatively: wound infection (*n* = 3), cystitis (*n* = 2), DVT (*n* = 1)

Abbreviations: vNOTES, vaginal natural-orifice transluminal endoscopic surgery; MPLS, multi-port laparoscopic surgery; BMI, body mass index; VAS, visual analog scale; SD, standard deviation; VANH, vaginal-assisted NOTES hysterectomy; BSO, bilateral salpingo-oophorectomy; SO, salpingo-oophorectomy; PFA, peritoneal fluid aspiration; SLN, sentinel lymph-node biopsy; DM, diabetes mellitus; HT, hypertension; OSA, obstructive sleep apnea; AF, atrial fibrillation; PAF, paroxysmal atrial fibrillation; DVT, deep-vein thrombosis. ^a^ Immobility of uterus (*n* = 2), high-risk features of endometrial cancer requiring laparoscopic lymph-node removal (*n* = 1), history of Crohn’s disease and the right ovary was tightly adherent to a redundant sigmoid colon (*n* = 1), a cystotomy occurred in the region of the trigone and required a urology consultation for repair (*n* = 1), a large uterus required mini-laparotomy for specimen removal (*n* = 1). ^b^ Bleeding of the ovarian vessels. ^c^ Patients underwent gynecological procedures for benign pathologies. ^d^ Patients underwent gynecological procedures for malignant pathologies. ^e^ Suboptimal vision (*n* = 3), unexpected endometriosis with adhesions to the side wall, adnexa, and sigmoid colon (*n* = 1), large bowel adhesions to the adnexa in a patient with a previous laparotomy (*n* = 1). ^f^ Impossibility of installing vNOTES ports (*n* = 2), inadequate adnexal exposure (*n* = 1), presence of dense pelvic adhesions (*n* = 1).

**Table 3:** Quality assessment of observational studies assessing vaginal natural-orifice transluminal endoscopic surgery (vNOTES) for gynecological procedures in obese patients, according to the Newcastle–Ottawa Scale (NOS).

First Author, Publication Year, Reference #	Selection	Comparability	Outcome	NOS Score
Wang F. et al., 2025 [17]	***	*	***	7
Bouchez M.-C. et al., 2023 [17]	***	*	**	6
Kaya C. et al., 2021 [15]	**	*	**	5
Matak L. et al., 2024 [18]	***	*	**	6

* = 1 point; ** = 2 points; *** = 3 points in each Newcastle-Ottawa Scale (NOS) domain (Selection, max 4; Comparability, max 2; Outcome, max 3). Total NOS score is the sum of domain points.

## Data Availability

The data used in this study are available upon request.

## Supplementary Materials

The following supporting information can be downloaded at: https://www.mdpi.com/article/10.3390/jcm14165713/s1, Table S1: (**a**) Preferred Reporting Items for Systematic Reviews and Meta-Analyses (PRISMA) 2020 Checklist. (**b**) Preferred Reporting Items for Systematic Reviews and Meta-Analyses (PRISMA) 2020 Abstracts Checklist.

## Abbreviations

The following abbreviations are used in this manuscript:

vNOTES	vaginal natural-orifice transluminal endoscopic surgery
PRISMA	Preferred Reporting Items for Systematic Review and Meta-analysis
NOS	Newcastle–Ottawa Scale
TLH	total laparoscopic hysterectomy
BMI	body mass index
VAS	visual analog scale
RCT	randomized controlled trial
WHO	World Health Organization
SD	standard deviation

## References

[1] Pickett C.M., Seeratan D.D., Mol B.W.J., Nieboer T.E., Johnson N., Bonestroo T., Aarts J.W. (2023). Surgical approach to hysterectomy for benign gynaecological disease. Cochrane Database Syst. Rev..

[2] Garry R. (2005). Health economics of hysterectomy. Best Pract. Res. Clin. Obs. Gynaecol..

[3] Baekelandt J., De Mulder P.A., Le Roy I., Mathieu C., Laenen A., Enzlin P., Weyers S., Mol B.W., Bosteels J.J. (2017). Postoperative outcomes and quality of life following hysterectomy by natural orifice transluminal endoscopic surgery (NOTES) compared to laparoscopy in women with a non-prolapsed uterus and benign gynaecological disease: A systematic review and meta-analysis. Eur. J. Obstet. Gynecol. Reprod. Biol..

[4] Kaya C., Yıldız Ş., Alay İ., Aslan Ö., Aydıner İ.E., Yaşar L. (2022). The Comparison of Surgical Outcomes following Laparoscopic Hysterectomy and vNOTES Hysterectomy in Obese Patients. J. Investig. Surg..

[5] Nesargikar P.N., Jaunoo S.S. (2009). Natural orifice translumenal endoscopic surgery (N.O.T.E.S). Int. J. Surg..

[6] Kale A., Sarıibrahim B., Başol G. (2017). Hysterectomy and salphingoopherectomy by Transvaginal Natural Orifice Transluminal Endoscopic Surgery(NOTES): Turkish surgeons’ initial experience. Int. J. Surg..

[7] Baekelandt J., De Mulder P., Le Roy I., Mathieu C., Laenen A., Enzlin P., Weyers S., Mol B.W.J., Bosteels J. (2019). Hysterectomy by transvaginal natural orifice transluminal endoscopic surgery versus laparoscopy as a day-care procedure: A randomised controlled trial. BJOG Int. J. Obstet. Gynaecol..

[8] Emmerich S., Fryar C., Stierman B., Ogden C. (2024). Obesity and Severe Obesity Prevalence in Adults: United States, August 2021–August 2023.

[9] Kahan S., Winston G. (2018). Addressing Obesity in Clinical Gynecology Practice. Clin. Obs. Gynecol..

[10] Matanes E., Eisenberg N., Amajoud Z., Gupta V., Yasmeen A., Ismail S., Racovitan F., Raban O., Lau S., Salvador S. (2021). Sentinel Lymph Node Sampling as an Alternative to Lymphadenectomy in Patients with Endometrial Cancer and Obesity. J. Obstet. Gynaecol. Can..

[11] McMahon M.D., Scott D.M., Saks E., Tower A., Raker C.A., Matteson K.A. (2014). Impact of Obesity on Outcomes of Hysterectomy. J. Minim. Invasive Gynecol..

[12] Grieco D.L., Anzellotti G.M., Russo A., Bongiovanni F., Costantini B., D’iNdinosante M., Varone F., Cavallaro F., Tortorella L., Polidori L. (2019). Airway Closure during Surgical Pneumoperitoneum in Obese Patients. Anesthesiology.

[13] Page M.J., McKenzie J.E., Bossuyt P.M., Boutron I., Hoffmann T.C., Mulrow C.D., Shamseer L., Tetzlaff J.M., Akl E.A., Brennan S.E. (2021). The PRISMA 2020 statement: An updated guideline for reporting systematic reviews. Int. J. Surg..

[14] Stang A. (2010). Critical evaluation of the Newcastle-Ottawa scale for the assessment of the quality of nonrandomized studies in meta-analyses. Eur. J. Epidemiol..

[15] Kaya C., Alay I., Cengiz H., Yıldız G.O., Baghaki H.S., Yasar L. (2021). Comparison of hysterectomy cases performed via conventional laparoscopy or vaginally assisted natural orifice transluminal endoscopic surgery: A paired sample cross-sectional study. J. Obs. Gynaecol..

[16] Bouchez M.C., Delporte V., Delplanque S., Leroy M., Vandendriessche D., Rubod C., Cosson M., Giraudet G. (2023). vNOTES Hysterectomy: What about Obese Patients?. J. Minim. Invasive Gynecol..

[17] Wang F., Liu Y., Xing Y., Wang D., Bai X., Li L., Ma C., Sun Y., Bai Y., Wang L. (2025). Clinical efficacy and safety study of vNOTES for benign ovarian tumors in obese patients. Sci. Rep..

[18] Matak L., Medić F., Sonicki Z., Matak M., Šimičević M., Baekelandt J. (2024). Retrospective analysis between total laparoscopic and vNOTES hysterectomy in obese patients: Single-center study. Arch. Gynecol. Obs..

[19] Mat E., Kale A., Gundogdu E.C., Basol G., Yildiz G., Usta T. (2021). Transvaginal natural orifice endoscopic surgery for extremely obese patients with early-stage endometrial cancer. J. Obstet. Gynaecol. Res..

[20] Burnett A.F., Pitman T.C., Baekelandt J.F. (2023). vNOTES (vaginal natural orifice transluminal surgery) gynecologic procedures in morbidly and super-morbidly obese women: Five year experience. Arch. Gynecol. Obs..

[21] Altintas M.M., Kuru B., Küçük H.F., Kaya S., Mat E., Cevik A. (2022). Concurrent hysterectomy and umbilical hernia repair via transvaginal notes among morbidly obese patients. Clin. Exp. Obs. Gynecol..

[22] Guevara R., Ortega C., Fernandez-Gonzalez S., Barahona M., Martinez J.M., Perez S., Torrejón-Becerra J.C., Castilla M., Alemany J., Cañizares A. (2024). 191 VNOTES feasibility in the surgical treatment of endometrial cancer: A case series. Int. J. Gynecol. Cancer.

[23] Kale A., Mat E., Başol G., Gündoğdu E.C., Aboalhasan Y., Yildiz G., Kuru B., Kale E., Usta T., Altıntaş M. (2022). A New and Alternative Route: Transvaginal Natural Orifice Transluminal Endoscopic Scarless Surgery (vaginal natural orifice transluminal endoscopic surgery) for Class 2 and Class 3 Obese Patients Suffering from Benign and Malignant Gynecologic Pathologies. Surg. Innov..

[24] Kapurubandara S., Baekelandt J., Laws P., King J. (2025). Adoption of vaginally assisted natural orifice transluminal endoscopic surgery for hysterectomy: A single tertiary experience. Aust. N. Z. J. Obstet. Gynaecol..

[25] Matak L., Šimičević M., Dukić B., Matak M., Baekelandt J. (2024). vNOTES surgical staging for endometrial carcinoma in overweight patients: A case series. Arch. Gynecol. Obs..

[26] Ng W., Lim N., Ang J.X., Wong Y.W.Y., Nadarajah R. (2024). Transvaginal natural orifice transluminal endoscopic surgery hysterectomy in patients with body mass index >50: An Asian experience. J. Obstet. Gynaecol. Res..

[27] Hurni Y., Simonson C., Di Serio M., Lachat R., Bodenmann P., Seidler S., Huber D. (2023). Feasibility and safety of vNOTES for gynecological procedures in obese patients. J. Gynecol. Obs. Hum. Reprod..

[28] Musin I.I., Berg E.A., Yashchuk A.G., Murtazina G.K., Ovsiuk D.N., Gimaeva Z.T. (2024). Vaginal natural orifice transluminal endoscopic total hysterectomy (vNOTES). Gynecol. Obstet. Clin. Med..

[29] Nef J., Hurni Y., Simonson C., Fournier I., Di Serio M., Lachat R., Bodenmann P., Seidler S., Huber D. (2025). Safety and efficacy of transvaginal natural orifice endoscopic surgery (vNOTES) for gynecologic procedures in the elderly: A case series of 119 consecutive patients. Eur. J. Obstet. Gynecol. Reprod. Biol..

[30] Housmans S., Noori N., Kapurubandara S., Bosteels J.J.A., Cattani L., Alkatout I., Deprest J., Baekelandt J. (2020). Systematic Review and Meta-Analysis on Hysterectomy by Vaginal Natural Orifice Transluminal Endoscopic Surgery (vNOTES) Compared to Laparoscopic Hysterectomy for Benign Indications. J. Clin. Med..

[31] Chaccour C., Giannini A., Golia D’Augè T., Ayed A., Allahqoli L., Alkatout I., Laganà A.S., Chiantera V., D’ORia O., Sleiman Z. (2023). Hysterectomy Using Vaginal Natural Orifice Transluminal Endoscopic Surgery Compared with Classic Laparoscopic Hysterectomy: A New Advantageous Approach? A Systematic Review on Surgical Outcomes. Gynecol. Obs. Investig..

[32] Yang E., Nie D., Li Z. (2019). Comparison of Major Clinical Outcomes Between Transvaginal NOTES and Traditional Laparoscopic Surgery: A Systematic Review and Meta-analysis. J. Surg. Res..

[33] Steinemann D.C., Müller P.C., Probst P., Schwarz A., Büchler M.W., Müller-Stich B.P., Linke G.R. (2017). Meta-analysis of hybrid natural-orifice transluminal endoscopic surgery versus laparoscopic surgery. Br. J. Surg..

[34] Lee C.L., Wu K.Y., Tsao F.Y., Huang C.-Y., Han C.-M., Yen C.-F., Huang K.-G. (2014). Natural orifice transvaginal endoscopic surgery for endometrial cancer. Gynecol. Minim. Invasive Ther..

[35] Oh S.H., Park S.J., Lee E.J., Yim G.W., Kim H.S. (2019). Pelvic lymphadenectomy by vaginal natural orifice transluminal endoscopic surgery (vNOTES) for early-stage endometrial cancer. Gynecol. Oncol..

[36] Tantitamit T., Lee C.L. (2019). Application of Sentinel Lymph Node Technique to Transvaginal Natural Orifices Transluminal Endoscopic Surgery in Endometrial Cancer. J. Minim. Invasive Gynecol..

[37] Huber D., Hurni Y. (2022). Sentinel Node Biopsy for Endometrial Cancer by Retroperitoneal Transvaginal Natural Orifice Transluminal Endoscopic Surgery: A Preliminary Study. Front. Surg..

[38] Baekelandt J.F. (2019). New Retroperitoneal Transvaginal Natural Orifice Transluminal Endoscopic Surgery Approach to Sentinel Node for Endometrial Cancer: A Demonstration Video. J. Minim. Invasive Gynecol..

[39] Deng L., Liu Y., Yao Y., Deng Y., Tang S., Sun L., Wang Y. (2023). Efficacy of vaginal natural orifice transluminal endoscopic sentinel lymph node biopsy for endometrial cancer: A prospective multicenter cohort study. Int. J. Surg..

[40] Baekelandt J., Kapurubandara S. (2021). Benign Gynaecological procedures by vaginal Natural Orifice Transluminal Endoscopic Surgery (vNOTES): Complication data from a series of 1000 patients. Eur. J. Obstet. Gynecol. Reprod. Biol..

[41] Marchand G.J., Ulibarri H., Arroyo A., Blanco M., Herrera D.G., Hamilton B., Ruffley K., Azadi A. (2024). Systematic review and meta-analysis of vaginal natural orifice transluminal endoscopic surgery hysterectomy versus vaginal hysterectomy for benign indications. AJOG Glob. Rep..

